# A new species of *Arachnanthus* from the Red Sea (Cnidaria, Ceriantharia)

**DOI:** 10.3897/zookeys.748.22914

**Published:** 2018-04-04

**Authors:** Sérgio N. Stampar, Suraia O. El Didi, Gustav Paulay, Michael L. Berumen

**Affiliations:** 1 Faculdade de Ciências e Letras, UNESP – Univ Estadual Paulista, Assis, Departamento de Ciências Biológicas, Laboratório de Evolução e Diversidade Aquática – LEDA, Av. Dom Antonio, 2100, Assis, SP, 19806-900, Brazil; 2 Florida Museum of Natural History, University of Florida, Gainesville FL 32611-7800, United States of America; 3 Red Sea Research Center, Division of Biological and Environmental Science and Engineering, King Abdullah University of Science and Technology, Thuwal, 23955-6900, Saudi Arabia

**Keywords:** Anthozoa, biodiversity, coral reefs, Indo-West Pacific, marine invertebrates, taxonomy

## Abstract

A new species of the genus *Arachnanthus* (Cnidaria: Ceriantharia), *Arachnanthus lilith* Stampar & El Didi, **sp. n.**, is described. This species is widely distributed in the Red Sea, and recorded from 2–30 m depths. *Arachnanthus lilith* Stampar & El Didi, **sp. n.** is the fifth species of the genus and the first recorded from the Red Sea. The number of labial tentacle pseudocycles, arrangement of mesenteries, and distribution of acontioids allow the differentiation of the new species from other species of the genus.

## Introduction

While tube anemones are common objects for underwater photographers and are widely exhibited in aquaria, they remain undersampled in most regions of the world, and the diversity and distribution of species remains poorly documented (Stampar et al. 2016). This is especially true for species that are difficult to observe and collect, because of nocturnal habits, small body size, or deeply extended burrows. The small, nocturnal tube anemones of the family Arachnactidae are a case in point (den Hartog 1977; Stampar et al. 2012, 2015a). This family is comprised of two benthic genera, *Arachnanthus* Carlgren, 1912 and *Isarachnanthus* Carlgren, 1924 (Stampar et al. 2016), although other genera have been proposed based only on larval forms (Molodtsova 2004). However, larval genera are not currently linked to those of adults and therefore their status remains unclear (Stampar et al. 2015a). Carlgren (1912) established *Arachnanthus* for *A. sarsi* (which he described from the North Sea) together with *Cerianthus oligopodus* Cerfontaine, 1891 from the Mediterranean. Carlgren (1924, 1937) later described *A. bockii* Carlgren, 1924 from Fiji and *A. australiae* Carlgren, 1937 from Australia. Since these studies, the genus has received little attention, with Picton and Manuel’s (1985) study and redescription of *A. sarsi* being the most substantive. Here a fifth species of *Arachnanthus* is described, the first known from Red Sea.

## Materials and methods

**Figure 1.**
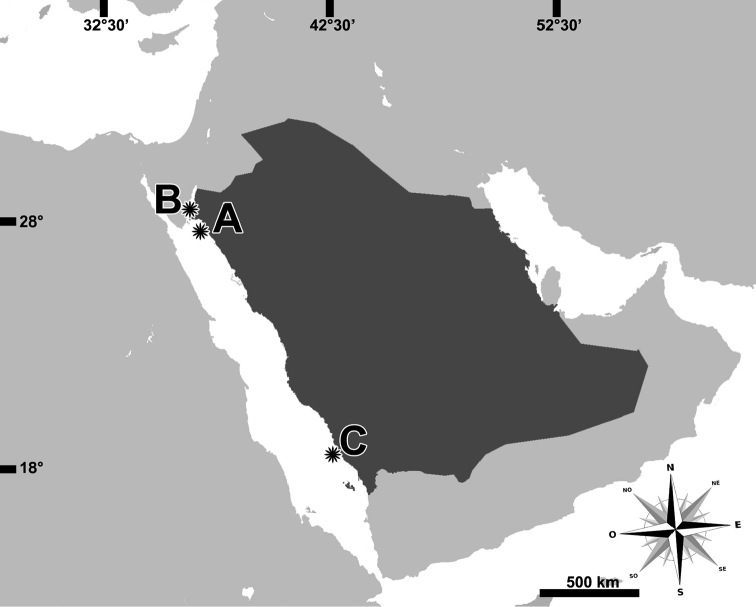
Records of *Arachnanthus lilith* sp. n. individuals studied, collected in Saudi Arabia (dark gray). A – UF Cnidaria 9168 (Holotype), B – UF Cnidaria 9167, UF Cnidaria 9227, UF Cnidaria 9229, UF Cnidaria 9230 (Paratype) and C – UF Cnidaria 9076.

Specimens were collected by hand at three sites across the Red Sea, from the Gulf of Aqaba to the Farasan Islands, in Saudi Arabia (Fig. 1). Collected polyps were preserved in 10 % buffered seawater formaldehyde solution, and later transferred to 75 % ethanol. The holotype and five paratypes are deposited in the Invertebrate Collections of the Florida Museum of Natural History, University of Florida (UF Cnidaria).

The anatomical study of polyps and cnidome were based on characters defined by previous authors (Carlgren 1912; den Hartog 1977; Stampar et al. 2012, 2015b). Six specimens were opened along the ventral side (opposite the siphonoglyph), using surgical scalpels, for anatomical study.

The classification of cnidae follows England (1991) and Stampar et al. (2015b). Thirty undischarged capsules were measured for each cnida type, sampled from each body region of two specimens (UF Cnidaria 9168 & 9229). The cnidome was studied with a Nikon Eclipse E200 microscope at 1000x magnification. Each part of the body was analyzed separately to avoid any contamination.

## Systematics

### Class Anthozoa Ehrenberg, 1834

#### Subclass Ceriantharia Perrier, 1883 (*sensu* Stampar et al. 2014)

##### Suborder Penicillaria den Hartog, 1977

###### Family Arachnactidae Carlgren, 1912

####### Genus Arachnanthus

Taxon classificationAnimaliaCerianthariaArachnactidae

Carlgren, 1912

######## Diagnosis.

Arachnactidae with sterile protomesenteries; metamesenteries in duplets (M and B), long (‘M’) metamesenteries with gonads and a double mesenteric filament, short (B) betamesenteries sterile, with single, convoluted mesenteric filament; very long stomodeum; lacking a directive labial tentacle; cnidome with p-mastigophores and b-mastigophores (after Carlgren 1912, 1924, 1937 and den Hartog 1977).

######## Type species.

*Arachnanthus oligopodus* (Cerfontaine, 1891)

######## Valid species

***Arachnanthus australiae* Carlgren, 1937**

***Arachnanthus bockii* Carlgren, 1924**

***Arachnanthus oligopodus* (Cerfontaine, 1891)**

***Arachnanthus sarsi* Carlgren, 1912**

***Arachnanthus lilith* sp. n.**

######## Distribution.

North Sea, Mediterranean Sea, Red Sea, East Australia, and Melanesia.

####### Arachnanthus lilith

Taxon classificationAnimaliaCerianthariaArachnactidae

Stampar & El Didi sp. n.

http://zoobank.org/FC381C67-9DB8-4280-9C9C-00DBD04F7D56

**Figure 2.**
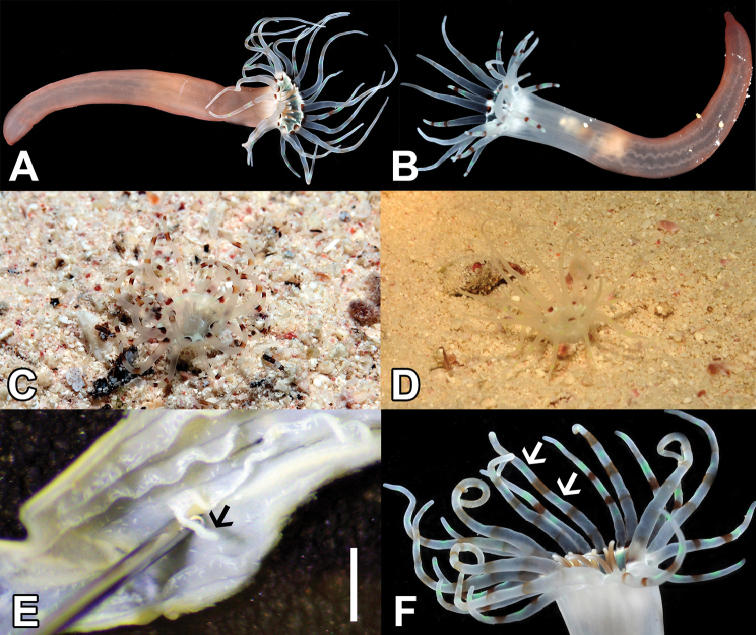
*Arachnanthus lilith* sp. n. **A** (Paratype UF Cnidaria 9227) (not to scale) **B** (Paratype UF Cnidaria 9168) (not to scale) **C–D** Live specimens in nature (not included as paratypes – ICZN 72.4.6) (not to scale) **E** Dissected specimen with detail of acontioids (arrows) (scale bar 2 mm) UF Cnidaria 9168 (Holotype) **F** Detail of oral disc UF Cnidaria 9229 (Paratype) with detail on tentacular pores with green fluorescent protein (GFP) (arrows) (not to scale).

**Figure 3.**
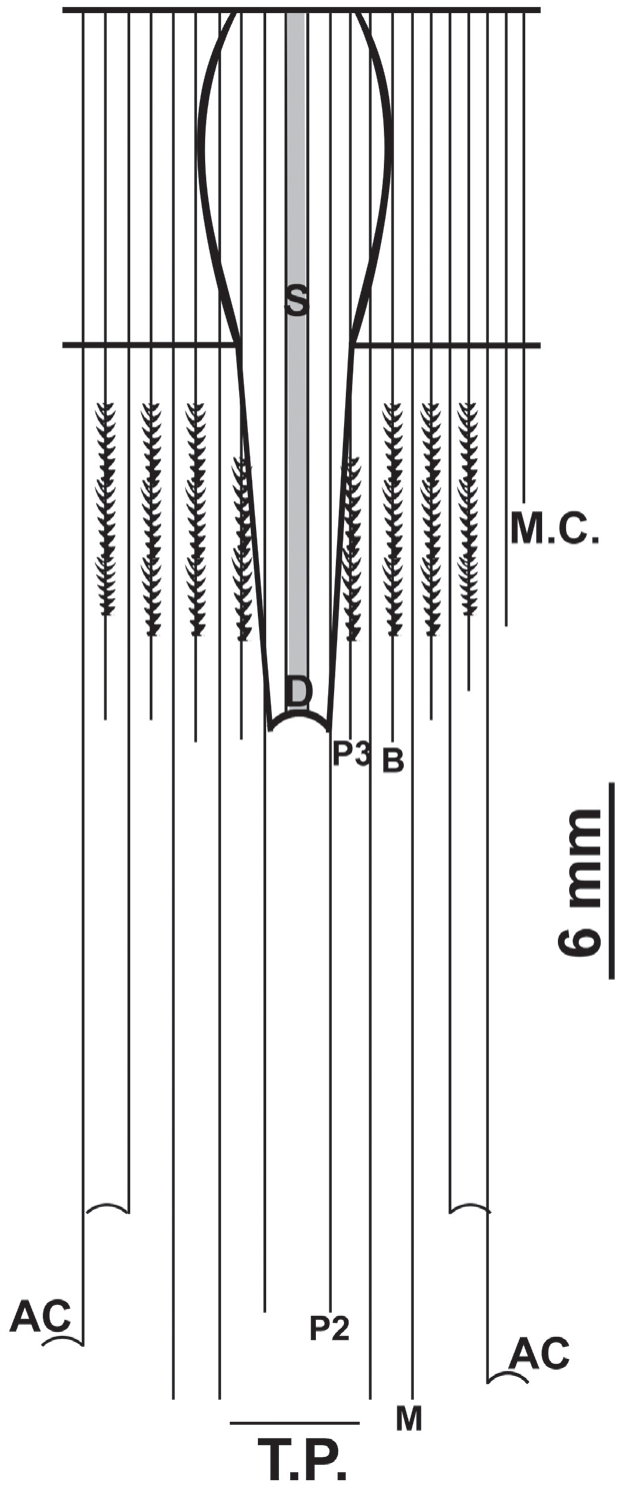
Graphical representation of the arrangement of mesenteries of *Arachnanthus lilith* sp. n. Abbreviations: M.C. multiplication chamber, D directives, T.P. terminal pore, S siphonoglyph, B betamesenteries (convoluted mesentery), M metamesenteries (double filament), P protomesenteries, AC acontioids

**Figure 4.**
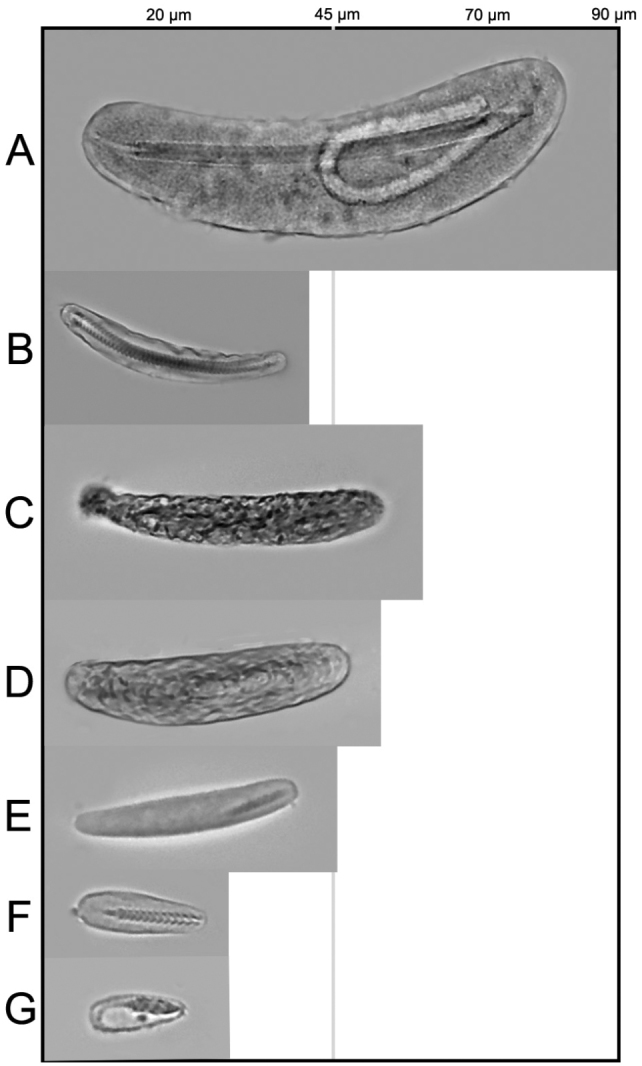
Cnidome of *Arachnanthus lilith* sp. n. **A** microbasic *p*-mastigophores I **B** microbasic *p*-mastigophores II **C** Atrich **D** Ptychocyst **E** microbasic *b*-mastigophores I **F** microbasic *b*-mastigophores II **G** microbasic *b*-mastigophores III.

Figs 1 , 2 , 3 , 4 , Tables 1 , 2

######## Material examined (six specimens).

**Holotype**: UF Cnidaria 9168, adult individual (35 mm long), Saudi Arabia, island near Jaz’air Sila, (27.651°N, 35.2832°E) (Fig. 1A), 10–30 m depth, fore reef, under rocks, G. Paulay, Seabird McKeon, Daisuke Uyeno coll. (27/ix/2013). **Paratypes**: UF Cnidaria 9167, adult (31 mm long), same data as holotype. UF Cnidaria 9227, adult (35 mm long), UF Cnidaria 9229, adult (42 mm long), UF Cnidaria 9230, adult (26 mm long) all three from Saudi Arabia, Gulf of Aqaba, Joey’s Shipwreck Bay, (28.1846°N, 34.6381°E) (Fig. 1B), 7–13 m depth, in sand and seagrass bed, collected at night, G. Paulay, Daisuke Uyeno, Casey Zakroff coll. (01/x/2013). UF Cnidaria 9076 (Fig. 2D), adult, Saudi Arabia, Farasan Banks, Atlantis Shoal (18.1917°N, 41.1138°E) (Fig. 1–C), 9–11 m depth, sandy shoal with patch reefs, in sand, collected at night, Arthur Anker, Patrick Norby, Gustav Paulay coll. (07/iii/2013).

######## Diagnosis.

Small ceriantharian, up to at least 42 mm long, 4–6 mm wide. With 19–24 translucent marginal tentacles (3–5 mm long in preserved specimens), each with 2–4 brown bands (Fig. 1); tentacle arrangement *(1)2.12.12.12.12*…; at least 5 pores per tentacle, pores marked by concentration of green fluorescent protein (GFP) (Fig. 1–F); unpaired marginal tentacle present. With 11–15 pale labial tentacles (up to 2 mm long in preserved specimens), tentacle arrangement *(0)3.12.31.23.23.12*…; unpaired labial tentacle absent. Long actinopharynx extending over 1/3 of total body length, hyposulcus 3–4 mm long, hemisulci distinct; siphonoglyph wide, connected to eight mesenteries; directive mesenteries a little shorter than hyposulcus. Three pairs of protomesenteries (P), P2 and P4 long and P3 short, metamesenteries (M), long, fertile with double mesenteric filament; betamesenteries (B) short, sterile with single mesenteric filament (double in a short part immediately below actinophrarynx) and rather convoluted; acontioids only in mesenteries M3 and M4; see Fig. 2 for schematic arrangement of mesenteries. Cnidome (Fig. 3) of spirocysts, atrichs, microbasic *b*-mastigophores (three types), microbasic *p*-mastigophores (two types), and ptychocysts; distributed as shown in Table 1.

**Table 1.** Cnidome of *Arachnanthus lilith* sp. n. based on two specimens (UF 9229; 9168). Mean and range given for each cnida.

		Length (in µm)	Width (in µm)
**Column**	Pytchocysts	**40.69** (37.7–44.2)	**9.18** (9.1–10.4)
	Atrichs	**48.57** (41.6–53.3)	**8.01** (6.5–10.4)
	b-mastigophores I	**32.32** (31.2–33.8)	**4.11** (3.9–5.2)
	p-mastigophores I	**86.45** (83.2–89.7)	**21.49** (19.5–23.4)
**Marginal tentacles**	p-mastigophores I	**84.15** (78.0–91.0)	**19.84** (18.2–20.8)
	p-mastigophores II	**33.75** (31.2–37.7)	**6.84** (6.5–7.8)
	b-mastigophores I	**32.63** (31.2–33.8)	**3.9** (3.8–4.0)
	b-mastigophores II	**21.06** (19.5–27.3)	**4.11** (3.9–5.2)
	Atrichs	**34.92** (31.2–39.0)	**6.58** (5.2–7.8)
**Labial tentacles**	p-mastigophores I	**64.87** (61.1–67.6)	**13.08** (11.7–14.3)
	b-mastigophores II	**25.3** (20.8–28.6)	**5.07** (3.9–7.8)
	Atrichs	**25.69** (24.7–28.6)	**6.02** (5.2–6.5)
**Stomodeum**	p-mastigophores I	**49.44** (45.5–54.6)	**9.83** (7.8–11.7)
	b-mastigophores II	**24.05** (20.8–26.0)	**5.76** (5.2–6.5)
	Atrichs	**33.28** (31.2–35.1)	**6.54** (5.2–7.8)
**Betamesenteries**	p-mastigophores I	**83.8** (80.6–89.7)	**23.14** (19.5–24.7)
	p-mastigophores II	**54.9** (52.0–58.5)	**15.34** (13.0–16.9)
	b-mastigophores II	**19.24** (15.6–23.4)	**4.03** (3.9–5.2)
**Metamesenteries**	b-mastigophores II	**25.04** (23.4–26.0)	**5.76** (5.2–6.5)
	b-mastigophores III	**17.76** (16.9–18.2)	**4.2** (3.9–5.2)

######## Distribution.

Presently known only from the Saudi Arabian Red Sea, from the Gulf of Aqaba to the Farasan Islands in the southern Red Sea. The species was found extended only at night.

######## Etymology.

The specific name *lilith* refers to the mythological figure of a female night demon in the vicinity of the Red Sea to ancient Mesopotamia (Saudi Arabia to Iraq).

######## Live color.

Column pinkish tan at basal half or along most of its length, becoming clear toward base of tentacles. Marginal tentacles whitish/transparent, with brown and light green bands; extent of banding variable, with a basal brown band commonly developed. Labial tentacles clear to brown, with whitish base and tips. Oral disk with green and white colors.

######## Description of holotype

(UF Cnidaria 9168). Small polyp, 35 mm long, 4 mm in diameter just below the marginal tentacles, 3 mm diameter near aboral end. With 19 marginal tentacles arranged in two pseudocycles, each 4 mm long and 0.5 mm in diameter near base, tentacle arrangement *(1)2.12.12.12.12*…. With 12 labial tentacles, each ~1 mm long, brown with a white apical tip, directive labial tentacle absent, tentacle arrangement *(0)3.12.31.23.12*…. Oral disc 0.7 mm wide, actinopharynx 17 mm long, light beige to light brown, siphonoglyph wide and elongate with eight mesenteries attached, hyposulcus 9 mm long. Directive mesenteries shorter than actinopharynx. Protomesenteries as in diagnosis, M-mesenteries (M), long, fertile with a double mesenteric filament; B-mesenteries (B) short, sterile with single mesenteric filament (double in a short part immediately below actinopharynx) and rather convoluted; acontioids only in mesenteries M3 and M4.

######## Comparison with other members of the genus.

Although Fautin et al. (2007) suggested that morphology alone is insufficient to distinguish species of this genus, internal anatomical characters do actually separate all known species (Table 2). While there are cases of cryptic species among tube-dwelling anemones (Stampar et al. 2012), none are yet documented for *Arachnanthus*.

**Table 2.** Comparison of anatomical features of species of *Arachnanthus* (after Carlgren 1912b; Carlgren 1924; Carlgren 1937; Picton and Manuel 1985; this study).

	*A. australiae*	*A. bockii*	*A. oligopodus*	*A. sarsi*	*A. lilith* sp. n.
**Marginal tentacles**	Up to 40	Up to 30	~20	Up to 35	Up to 24
**Arrangement of labial tentacles**	(0)1.11.11.11.11	(0)1.11.11.11.11(?)	(0)1.11.11.11.11	(0)1.11.11.11.11	(0)3.12.31.23.23.12
**Length of actinopharynx**	~2/3 of gastric cavity	~1/2 of gastric cavity	~1/2 of gastric cavity	~1/2 of gastric cavity	>1/2 of gastric cavity
**Hyposulcus**	~1/2 size of stomodeum	~1/2 size of stomodeum	~2X size of stomodeum	< size of stomodeum	= size of stomodeum
**Oral disc diameter**	~0.7 cm	–	–	~1 cm	0.5 cm
**Maximum n° of mesentery attached to siphonoglyph**	12	12	4	6	8
**Directive mesenteries**	= length of Actinopharynx	< length of Actinopharynx	> length of Actinopharynx	< length of Actinopharynx	< length of Actinopharynx
**P(C)2**	Short, 1/2 of gastric cavity	Very short, 1/4 of gastric cavity	Short, 1/2 of gastric cavity	Long, 3/4 of gastric cavity	Long, 6/7 of gastric cavity, almost to aboral pole
**P(C)3**	Very short, <1/4 of gastric cavity	Very short, <1/4 of gastric cavity	Short, ~1/2 of gastric cavity	Short, ~1/3 of gastric cavity	Short, 1/3 of gastric cavity
**M1**	Almost to aboral pore	Almost to aboral pore	To aboral pore	Almost to aboral pore	To aboral pore
**M3**	4/5 of gastric cavity	Almost to aboral pore	1/5 of gastric cavity	Almost to aboral pore	3/4 of gastric cavity
**Cnido-glandular tract of fertile mesenteries**	Present (short?)	Present (short?)	Present	Present	Present
**Cnido-glandular tract of B**	Present (short?)	Present (short?)	Present (short?)	Present (short)	Present (short)
**Acontioids**	Only in M1, M2 and M3	Only in M1, M2 and M3	Only in M1	Only in M1, M2 and M3	Only in M3 and M4
**Distribution**	Northern Australia	Fiji	Mediterranean Sea	North Sea	Red Sea

*Arachnanthus lilith* has labial tentacles in three pseudocycles, unlike *A. australiae*, *A. oligopodus*, and *A. sarsi*, which all have them in one pseudocycle, while in *A. bockii* labial tentacles are not clearly organized and may be considered to fall into one or two pseudocycles. The actinopharynx is 2/3 as long as the gastric cavity in *A. australiae*, less than ½ as long in the other three described species, and a little over ½ as long in *A. lilith*. The maximum number of the mesenteries attached to the siphonoglyph is especially useful for distinguishing species: *A. australiae* and *A. bockii* have 12 each, *A. lilith* has eight, *A. sarsi* six, while *A. oligopodus* has four. The organization of mesenteries, particularly the mesentery P2 and M3, also provides useful characters to separate species (Table 2). Finally, the distribution of acontioids is also quite different in some species, especially in *A. lilith* where acontioids are present only on mesenteries M3 and M4. These mesenterial characters serve well to differentiate species of *Arachnanthus*, although how they vary over the ontogeny of each species remains to be studied.

Finally, the present study demonstrates the importance of more detailed investigations using non-standard collecting techniques. Small ceriantharians are rarely collected as they are frequently nocturnal and can be difficult to extract from the sediment as they retract quickly and rapidly. There are few described species of Ceriantharia with small body sizes; however, this may be the result of sampling limitations.

## Supplementary Material

XML Treatment for Arachnanthus

XML Treatment for Arachnanthus lilith
